# Interplay between natriuretic peptides and left atrial mechanics and the relation to recurrence of atrial fibrillation following catheter ablation

**DOI:** 10.1007/s10554-023-02913-y

**Published:** 2023-07-10

**Authors:** Flemming Javier Olsen, Stine Darkner, Jens Peter Goetze, Xu Chen, Kristoffer Henningsen, Steen Pehrson, Jesper Hastrup Svendsen, Tor Biering-Sørensen

**Affiliations:** 1 Department of Cardiology, Copenhagen University Hospital - Herlev and Gentofte, Hellerup, Denmark; 2 Department of Clinical Biochemistry, Copenhagen University Hospital - Rigshospitalet, Copenhagen, Denmark; 3 Department of Cardiology, Copenhagen University Hospital - Rigshospitalet, Copenhagen, Denmark; 4 Department of Clinical Medicine, University of Copenhagen, Copenhagen, Denmark; 5 Department of Biomedical Sciences, University of Copenhagen, Copenhagen, Denmark; 6 Cardiovascular Non-Invasive Imaging Research Laboratory, Department of Cardiology, Copenhagen University Hospital - Herlev and Gentofte, Gentofte Hospitalsvej 1, 2900 Hellerup, Denmark

**Keywords:** Natriuretic peptides, Atrial fibrillation, Left atrium, Echocardiography

## Abstract

The relationship between natriuretic peptides and atrial distension is not fully understood. We sought to examine their interrelationship and how they relate to atrial fibrillation (AF) recurrence following catheter ablation. We analyzed patients enrolled in the AMIO-CAT trial (amiodarone vs. placebo for reducing AF recurrence). Echocardiography and natriuretic peptides were assessed at baseline. Natriuretic peptides included mid-regional proANP (MR-proANP) and N-terminal proBNP (NT-proBNP). Atrial distension was assessed by left atrial strain measured by echocardiography. The endpoint was AF recurrence within 6 months after a 3-month blanking period. Logistic regression was used to assess the association between log-transformed natriuretic peptides and AF. Multivariable adjustments were made for age, gender, randomization, and left ventricular ejection fraction. Of 99 patients, 44 developed AF recurrence. No differences in natriuretic peptides nor echocardiography were observed between the outcome groups. In unadjusted analyses, neither MR-proANP nor NT-proBNP were significantly associated with AF recurrence [MR-proANP: OR = 1.06 (0.99–1.14), per 10% increase; NT-proBNP: OR = 1.01 (0.98–1.05), per 10% increase]. These findings were consistent after multivariable adjustments. However, atrial strain significantly modified the association between MR-proANP and AF (p for interaction = 0.009) such that MR-proANP was associated with AF in patients with high atrial strain [OR = 1.24 (1.06–1.46), p = 0.008, per 10% increase] but not in patients with low atrial strain. In patients with high atrial strain, an MR-proANP > 116 pmol/L posed a fivefold higher risk of AF recurrence [HR = 5.38 (2.19–13.22)]. Atrial natriuretic peptide predicts AF recurrence in patients with preserved atrial distension. Assessing atrial strain may assist the interpretation of natriuretic peptides.

**Graphical abstract:**

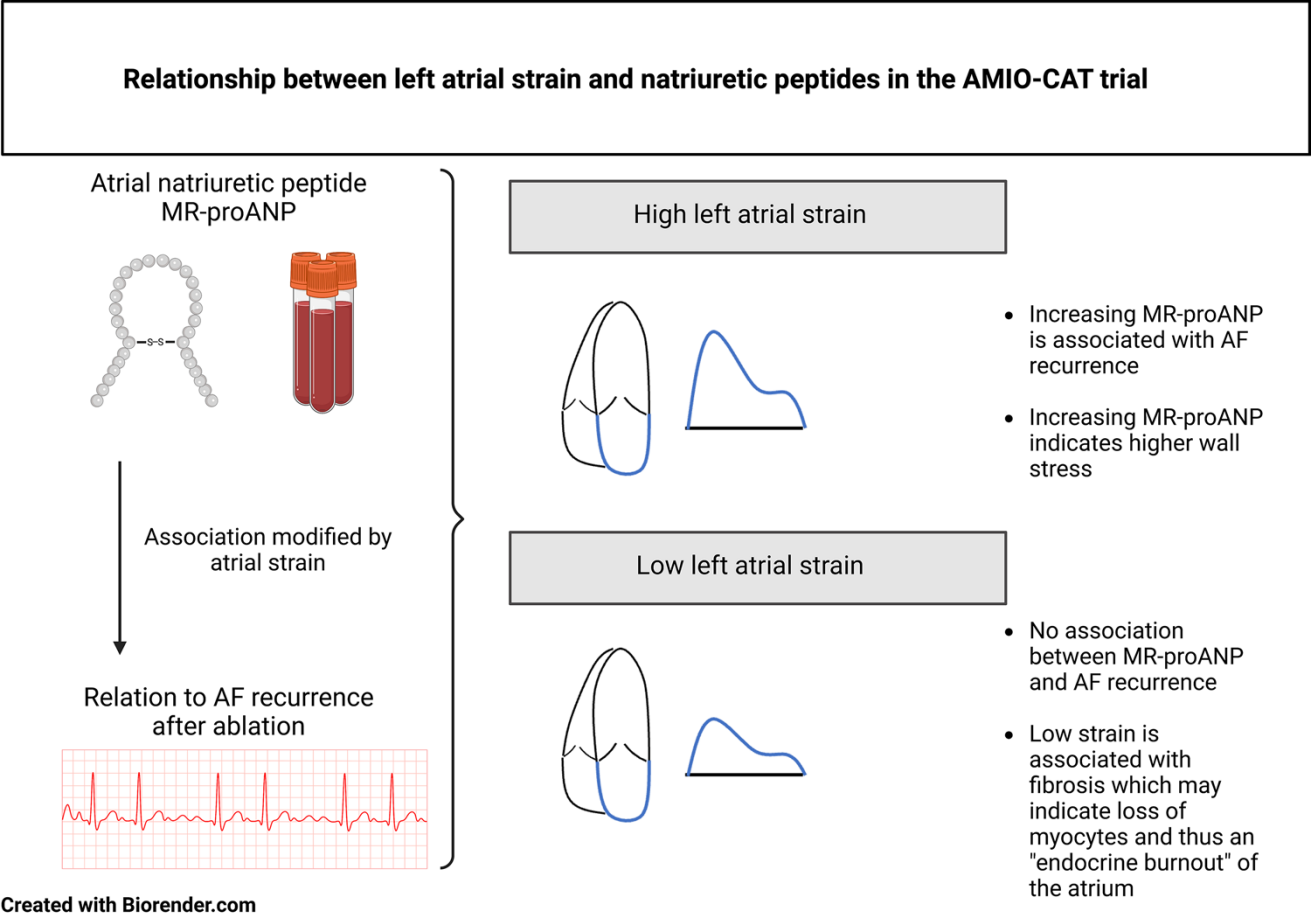

## Introduction

Globally, 38 million individuals are affected by atrial fibrillation (AF), an atrial arrhythmia that is responsible for approximately 300,000 deaths annually [1]. Catheter ablation represents an important treatment option for rhythm management in symptomatic AF [2]. Although effective, AF recurrence is frequently observed with some patients requiring multiple ablation attempts [3]. Establishing predictors of AF recurrence may improve clinical management, and biomarkers such as natriuretic peptides have been proposed as potential risk markers [4, 5]. Natriuretic peptides are secreted from myocytes in the atria in response to distension with the purpose of regulating systemic vascular resistance, natriuresis, and ultimately blood pressure through numerous pathways [6, 7]. Natriuretic peptides may therefore hold clinical value for diagnostic as well as prognostic purposes in atrial disease including AF. Even though elevated concentrations of natriuretic peptides have shown potential for detecting paroxysmal AF [8], conflicting results have been observed when it comes to predicting AF recurrence following catheter ablation [4, 9]. The reasons for these conflicting results may stem from the complicated nature of how natriuretic peptides are secreted with progressive left atrial (LA) mechanical dysfunction [10]. Hence, examining the inter-relationship between echocardiographic measures of LA distension and natriuretic peptides may be a sensible approach to assist the interpretation of natriuretic peptide concentration [7]. Similarly, applying LA mechanics may provide more detailed insights as to the relationship between natriuretic peptides and AF recurrence following ablation. To that end, the purpose of the present study was to examine the predictive value of natriuretic peptides in combination with echocardiographic measures of LA distensibility as assessed by two-dimensional volume quantification and LA speckle tracking.

## Methods

### Study population

This was a substudy of the randomized, double-blinded, placebo-controlled AMIO-CAT trial (Clinicaltrials.gov ID: NCT00826826) which sought to investigate whether short-term amiodarone treatment reduced AF recurrence following catheter ablation. A detailed description of the trial has previously been outlined [11].

This substudy only included participants from Rigshospitalet (n = 124), one of two centers that participated in the main trial, as only this institution performed natriuretic peptide measurements as part of the study. These patients were enrolled from February 2009 to July 2013. Of the 124 patients enrolled at Rigshospitalet, 19 did not have natriuretic measurements performed, 3 were excluded as they had a creatinine > 150 µmol/L, 2 patients did not have Holter monitoring performed at 6-month follow-up, and 1 patient did not have any echocardiographic measures of the LA available, leaving 99 patients for final inclusion.

### Ethics

The study complied with the Helsinki Declaration, was approved by a regional scientific ethics committee, the Danish Data Protection Agency, the Danish Medicines Agency and informed consent was obtained from all participants.

### Endpoint

The endpoint was late AF recurrence defined as documented atrial tachyarrhythmia > 30 s after a 3-month blanking period. Patients had 72-h Holter monitoring performed at 6 months for rhythm monitoring.

### Natriuretic peptides

Patients had venous blood samples drawn at study inclusion, prior to ablation. N-terminal pro B-type natriuretic peptide (NT-proBNP) was measured on the automated Modular P platform (limit of detection ≥ 5.9 pmol/L). Mid-regional pro atrial natriuretic peptide (MR-proANP) was measured on the automated Kryptor Plus platform (Thermo Fisher, Hennigsdorf, Germany) after the plasma had been stored at − 80 °C. The intra-assay coefficient of variation according to the manufacturer is < 2.5% when the concentration is within the relevant range of 20 to 1000 pmol/L (limit of detection ≥ 2.1 pmol/L, and limit of quantitation ≥ 4.5 pmol/L).

### Echocardiography

Detailed description of the echocardiographic analyses have previously been described [12]. All patients had a transthoracic echocardiogram (Philips iE 33, Netherlands) performed prior to catheter ablation (0–90 days before). Echocardiographic measurements were performed using Xcelera quantification software (Philips Healthcare), with the exception of speckle tracking which was performed with Epsilon Imaging vendor-independent software (EchoInsight® software revision 2.2.0.x).

Left ventricular systolic function was evaluated by the left ventricular ejection fraction (LVEF) measured by the Simpson’s biplane method. LA volumes were measured by the biplane area-length method at end-systole and end-diastole. They were indexed to body surface area to provide the left atrial volume index (LAVi) and left atrial end-diastolic volume index (LAEDVi), respectively. The LA expansion index (LAi) was calculated as the fractional difference between these two volumes (LAVi − LAEDVi)/LAEDVi.

LA speckle tracking was performed by manual delineation along the endocardial border in the apical 4- and 2-chamber views after which the software generated a region of interest to cover the LA wall. The software subsequently provided corresponding strain values, including the peak LA reservoir strain. LA speckle tracking was feasible in 94% of these patients.

LAVi, LAi, and LA reservoir strain all represent measures of the reservoir/distension function of the LA [13].

### Statistics

Baseline characteristics are listed for the entire population and according to the outcome of AF recurrence. Differences between groups were tested by Student’s t-test for Gaussian distributed continuous variables and with Mann–Whitney U test for non-Gaussian distributed continuous. Categorical variables were compared by Chi^2^-test and Fisher’s exact test as appropriate. All continuous variables are expressed as mean values ± standard deviation, continuous non-parametric variables are presented by the median (IQR) and categorical variables as total numbers with percentages. Logistic regression was used to assess the risk of AF recurrence according to changes in natriuretic peptides. The natriuretic peptides were log-transformed for these analyses. Log-transformation was done in a log(1.10) approach that allowed for assessment of change in risk according to 10% changes in natriuretic peptide concentration. Multivariable adjustment was made for age, gender, LVEF, and randomization group. Test for effect modification was performed to assess whether randomization group (amiodarone treatment vs. placebo) modified the associations between natriuretic peptides and AF recurrence. Similarly, tests for interaction were performed for measures of LA distension (LA reservoir strain, LAi, and LAVi) to assess for any effect modification on the association between natriuretic peptides and AF recurrence.

A restricted cubic spline curve based on Poisson regression was created to visualize the continuous association between MR-proANP (untransformed) and risk of AF recurrence. The number of knots were chosen based on the lowest Akaike information criterion.

Kaplan–Meier curves were created to visualize the risk of AF recurrence at follow-up stratified by MR-proANP value (above vs. below the population median value of 116 pmol/L) in the patients with preserved LA strain.

For all analyses, a p-value ≤ 0.05 in two-tailed tests was considered statistically significant. STATA Statistics/Data analysis SE 15.1 (StataCorp, Texas, USA) was used for statistical analysis. Kaplan–Meier curves were created with R Studio (v. 3.6.0).

## Results

A total of 99 patients were included in this sub-study of whom 44 (44%) developed AF recurrence after the 3-month blanking period. Baseline characteristics for the entire population and stratified by outcome are shown in Table 1. Briefly, the mean age was 58 years, 82% of the participants were men, 45% had persistent AF, and mean LVEF was 51%. The median MR-proANP concentration was 116 pmol/L. There were no between group differences observed except for randomization. Likewise, there were no differences in natriuretic peptides nor echocardiographic characteristics between the two outcome groups.

**Table 1:** Characteristics according to outcome

	All n = 99	No AF recurrence n = 55	AF recurrence n = 44	P-value
Age (years)	58 ± 10	57 ± 9	59 ± 11	0.41
Men, n (%)	81 (82)	46 (84)	35 (80)	0.60
Randomization to amiodarone, n (%)	52 (53)	37 (67)	15 (34)	0.001
Body mass index (kg/m^2^)	27 ± 4	27 ± 4	27 ± 4	0.75
Heart rate, beats per minute	70 ± 18	71 ± 20	68 ± 15	0.53
Prior ablation, n (%)	31 (31)	19 (35)	12 (27)	0.44
Persistent AF, n (%)	45 (45)	27 (49)	18 (41)	0.42
Hypertension, n (%)	32 (32)	17 (31)	15 (34)	0.74
Diabetes mellitus, n (%)	10 (10)	5 (9)	5 (11)	0.71
Ischemic heart disease, n (%)	7 (7)	4 (7)	3 (7)	0.93
Prior ischemic stroke, n (%)	8 (8)	3 (5)	5 (11)	0.28
History of atrial flutter, n (%)	6 (6)	4 (7)	2 (5)	0.57
CHADS-score, n (%)				0.16
0	44 (44)	29 (53)	15 (34)	
1	23 (23)	10 (18)	13 (30)	
> 2	32 (32)	16 (29)	16 (36)	
MR-proANP (pmol/L)	116 [82; 179]	101 [67; 153]	125 [88; 192]	0.11
NT-proBNP (pmol/L)	20 [9; 64]	19 [8; 46]	21 [9; 69]	0.45
LVEF (%)	51 ± 9	50 ± 9	52 ± 9	0.26
LA volume (mL/m^2^)	36 ± 11	37 ± 12	36 ± 10	0.66
LA expansion index (%)	49 [28; 78]	52 [30; 83]	48 [25; 76]	0.40
LA reservoir strain (%)	24 ± 9	23 ± 9	24 ± 8	0.68

*AF* atrial fibrillation, *MR-proANP* mid-regional pro atrial natriuretic peptide, *NT-proBNP* N-terminal pro brain natriuretic peptide, *LVEF* left ventricular ejection fraction, *LA* left atrium

**Fig. 1:**
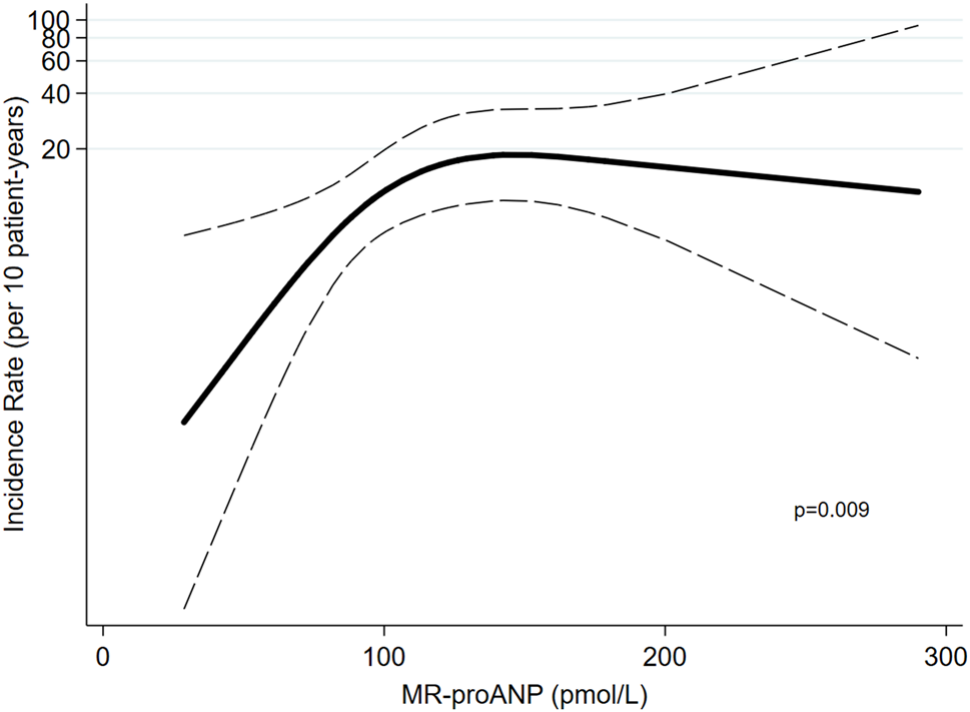
Continuous association between MR-proANP and AF. Restricted cubic spline curve shows a non-linear continuous association between MR-proANP and the incidence rate of AF in patients with high LA strain (> 23%). The risk increases steadily with increasing MR-proANP until MR-proANP reaches a level of approximately 140 pmol/L, after which the risk slightly decreases. *MR-proANP* mid-regional pro atrial natriuretic peptide, *AF* atrial fibrillation, *LA* left atrial

**Fig. 2:**
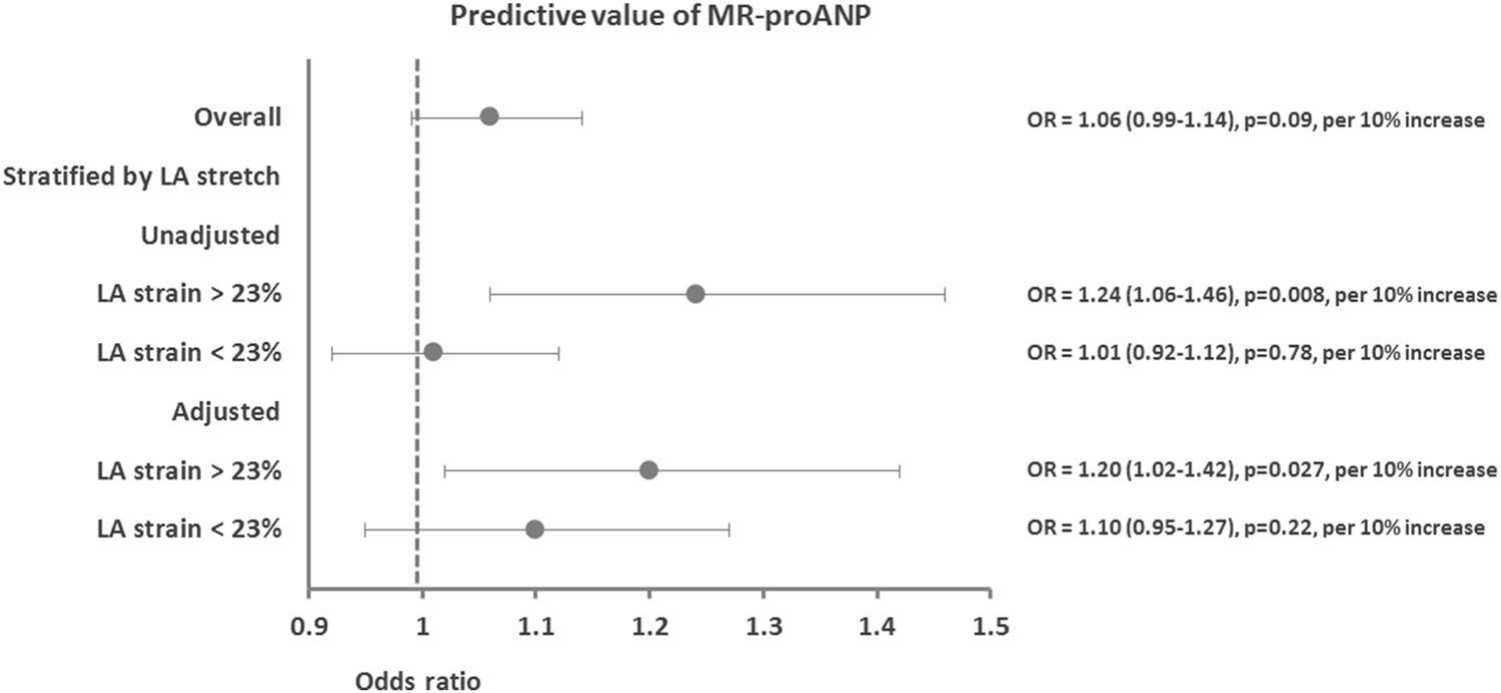
Predictive value of MR-proANP. Forest plot showing the association between MR-proANP and recurrence of AF after the 3-month blanking period. The upper panel shows the estimates for the entire population. The middle panel shows the unadjusted estimates stratified by high and low LA strain (cutoff: 23%). The lower panel shows the adjusted estimates stratified by high and low LA strain. Dots denote unadjusted estimates and error bars represent 95% confidence intervals. Adjustments were made for LVEF, age, gender, and randomization. *MR-proANP* mid-regional pro atrial natriuretic peptide, *LA* left atrial

In univariable logistic regression, MR-proANP was not associated with AF recurrence [OR = 1.06 (0.99–1.14), p = 0.09, per 10% increase], which was also the case for NT-proBNP [OR = 1.01 (0.98–1.05), p = 0.38, per 10% increase]. After multivariable adjustment, these findings were unchanged. However, test for effect modification revealed that LA strain significantly modified the association between MR-proANP and AF recurrence (p for interaction = 0.009) and showed a trend for effect-modification between NT-proBNP and AF as well (p for interaction = 0.058). No other measure of atrial distension (LAVi and LAi) significantly modified the associations between natriuretic peptides and AF recurrence (p for interaction > 0.05 for all). LA strain modified the association between MR-proANP and AF recurrence in such a way that increasing MR-proANP predicted AF recurrence in patients with high LA strain (above 23%) (n = 47, events: 20) but not in patients with low LA strain (n = 47, events: 21). In the subgroup with high LA strain, the risk of AF recurrence increased steadily with increasing MR-proANP until it reached a level of approximately 140 pmol/L, after which the risk decreased slightly (Fig. 1). Overall, for patients with high LA strain, a 10% increase in MR-proANP was associated with a 24% higher likelihood of AF recurrence in unadjusted analyses [OR = 1.24 (1.06–1.46), p = 0.008, per 10% increase]. These findings were consistent after multivariable adjustments (Fig. 2). In contrast, NT-proBNP was not associated with AF recurrence for neither those with high nor low LA strain [NT-proBNP for those with low LA strain: OR = 1.01 (0.97–1.06), p = 0.66, and for high LA strain: 1.05 (0.97–1.13), p = 0.21, per 10% increase in NT-proBNP].

**Fig. 3:**
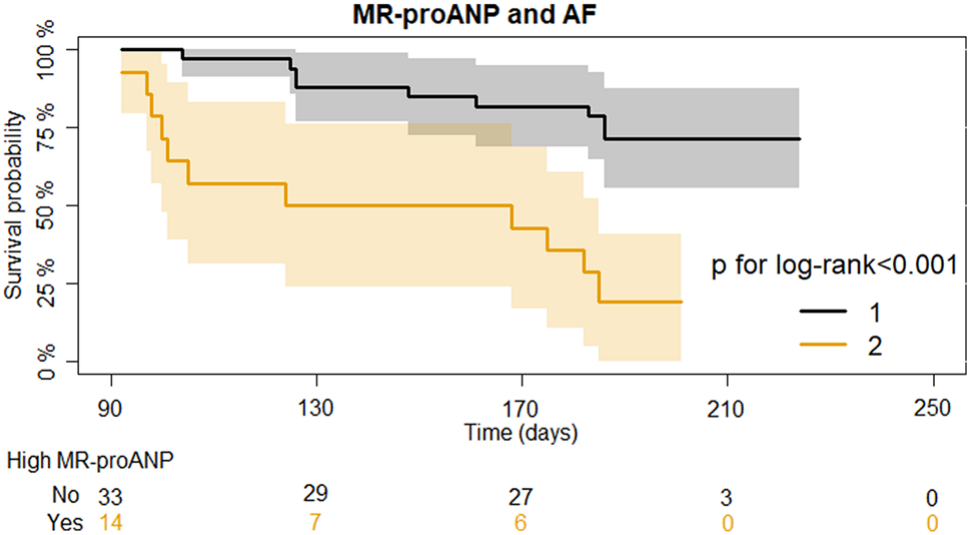
Risk of AF according to MR-proANP concentration. Kaplan–Meier estimates for developing AF recurrence in the subset of patients with a high left atrial strain stratified by high vs. low MR-proANP (defined by population median value of 116 pmol/L). *AF* atrial fibrillation, *MR-proANP* mid-regional pro atrial natriuretic peptide

In the subgroup of patients with high LA strain (n = 47), those with a MR-proANP > 116 pmol/L had a more than fivefold higher risk of AF recurrence as compared to those with MR-proANP below the median value of 116 pmol/L [HR = 5.38 (2.19–13.22), p < 0.001]. Kaplan–Meier estimators of late AF recurrence during follow-up stratified by high vs. low MR-proANP are shown in Fig. 3.

## Discussion

The principal finding of the present study was that LA distension modified the association between MR-proANP and AF recurrence. Adding LA strain provides a more nuanced assessment of patients at risk. It also provides an example of how LA mechanics as assessed by echocardiography can be used to improve our understanding and the clinical applications of natriuretic peptides.

### Natriuretic peptides and AF recurrence

As 30–50% of patients develop AF recurrence following first catheter ablation [3], there is a continued need for optimizing the selection of patients to identify those who will not benefit at all from ablation treatment—to prevent unnecessary procedures—as well as those who will likely need re-ablation and therefore require close monitoring following their first ablation. A meta-analysis from Jiang et al. has previously established that plasma ANP can predict AF recurrence following catheter ablation [4]. This meta-analysis included six studies with a collective population of 298 patients and 113 recurrences. However, the studies were heterogeneous in design and included AF recurrence within the 3-month blanking period, during which AF events are generally not considered failure of catheter ablation [14]. When only considering studies with AF recurrence outside the blanking period, plasma ANP was not predictive of AF recurrence. The lack of predictive ability of plasma ANP has since been confirmed by Nakanishi [9], and similar results have been observed for other ANP precursors [15, 16]. Overall, the findings consistently show that ANP is not predictive of significant AF recurrence and stresses a need for exploring other ways to use ANP instead. So far, studies investigating the predictive value of natriuretic peptides have primarily operated under the assumption that increasing natriuretic peptide concentration translates into increased AF recurrence risk in a linear fashion. However, Daniels et al. outlined another way of considering the association between natriuretic peptide concentration and AF risk by linking the endocrine function to the atrial viability [10]. In patients without atrial fibrosis, a low natriuretic peptide concentration represents a healthy atrium and a high concentration of natriuretic peptide reflects pathology. In patients with extensive atrial fibrosis the interpretation of natriuretic peptide concentration is more complicated as a low natriuretic peptide concentration may indicate an endocrine burnout due to degenerative changes [17, 18], and a low ANP concentration has indeed previously been shown to predict AF recurrence following cardioversion [19]. On the other hand, a high concentration in part reflects ongoing disease state but also that the atrium is healthy enough to produce a secretory response. This complex response in natriuretic peptide secretion with progressive mechanical dysfunction may explain why increasing atrial natriuretic peptide concentration failed to predict AF recurrence in patients with low LA strain. The reason why LA strain, as opposed to the other measures of LA distension, modified the association between natriuretic peptides and AF may be explained by the fact it is a direct measure of atrial tissue function. By extension, LA strain has been shown to be closely linked to atrial fibrosis [20], and may be therefore be used as a surrogate measure of atrial fibrosis.

## Limitations

The patients were included as part of a randomized controlled trial and thereby under strict selection criteria, which limits generalizability. We had a small sample size as only one of the two centers measured natriuretic peptide concentrations. This also means that our multivariable models may have suffered from overfitting in the subgroup analyses. Since the patients underwent 72-h Holter monitoring and did not have continuous rhythm monitoring performed, we may have missed some AF recurrences. However, this strategy reflected the clinical approach at the time of the study.

## Conclusion

Atrial natriuretic peptide predicts AF recurrence after the blanking period following catheter ablation, specifically in patients with preserved LA distension. NT-proBNP does not predict AF recurrence.
